# Impact of molecular diagnostics and targeted cancer therapy on patient outcomes (MODIFY): a retrospective study of the implementation of precision oncology

**DOI:** 10.1002/1878-0261.13785

**Published:** 2024-12-11

**Authors:** Michaël Dang, Anna Schritz, Nikolai Goncharenko, Guy Berchem

**Affiliations:** ^1^ Department of Oncology Centre Hospitalier de Luxembourg (CHL) Luxembourg; ^2^ University of Luxembourg (Uni.lu) Luxembourg; ^3^ Luxembourg Institute of Health (LIH) Luxembourg; ^4^ Institut National du Cancer (INC) Luxembourg Luxembourg

**Keywords:** genomic profiling, matched therapy, PFS ratio, precision oncology

## Abstract

High‐throughput genomic analyses are being implemented in clinical practice. MODIFY is a retrospective study of the first introduction of genomic profiling and molecular tumor boards in the country of Luxembourg. The primary objective was to assess whether patients derived a clinical benefit by measuring the percentage of patients who presented a progression‐free survival (PFS) on matched therapy (PFS2) 1.3‐fold longer than PFS on previous therapy (PFS1). A total of 94 patients were included. In total, 45 patients (53.57% of patients with successful next‐generation sequencing [NGS] analysis) were found to have an actionable mutation. Of these, 11 patients received the treatment recommended by the molecular tumor board, another 12 received best‐supportive care, and 20 were treated with conventional therapy. PFS2 and PFS1 data were available for eight patients. The PFS2/PFS1 ratio was ≥ −1.3 in 62.5% (*n* = 5/8; CI [30.38, 86.51]) of patients; three patients showed a partial response, and median overall survival (OS) was 7.3 months. Although the examined population was small, this study further supports evidence indicating that patients with advanced cancer benefit from molecular profiling and targeted therapy.

## Graphical abstract

The authors conducted a retrospective study of 94 patients with advanced cancer who underwent next‐generation sequencing (NGS) gene panel analysis and received targeted treatments when applicable. Results further support evidence indicating that molecular profiling provides clinical benefit.

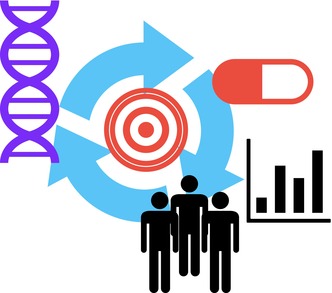

**Abbreviations**
CR: complete response
HRD: homologous recombination deficiency
MSI: microsatellite instability
MTB: molecular tumor board
NGS: next‐generation sequencing
OS: overall survival
PD: progressive disease
PFS: progression‐free survival
PR: partial response
SD: stable disease

## 1 Introduction

The use of molecular profiling to target oncogenic drivers has already had a profound effect on cancer therapy and patient outcomes even though implementation in daily practice has been an ongoing challenge. Targeted therapy of select molecular alterations has been recognized as improving outcomes in several entities [1, 2, 3] and has been established as standard‐of‐care, with an increasing number of emerging targetable tumor agnostic molecular biomarkers [4, 5]. However, as tumor characterization potential grows and the number of potential targets and biomarkers of resistance expands, translation into clinical practice requires a new precision oncology specific workflow. The establishment of evidence‐based hierarchical ranking scales of molecular alteration [6], clinical knowledgebases [7, 8, 9] to classify known genomic aberrations and dedicated molecular tumor boards to assess relevance of a given therapeutic option for individual patients, have allowed to facilitate this process of daily practice implementation. Guidelines and recommendations [10] based on growing experience have also emerged to help establish or improve dedicated workflow processes in this field.

Nevertheless, efficacy of precision oncology has also been hampered by an incomplete knowledge of biological resistance mechanisms [11, 12] such as activation of compensatory pathways for cell survival, drug target alteration [13], tumor microenvironment regulation [14], and intrinsic cancer heterogeneity [15]. As such, molecular tumor boards are needed to translate the existing preclinical and often early‐phase trial extracted knowledge into therapeutic recommendations, which have a potential benefit for patients with advanced solid tumors who have often undergone multiple therapy lines.

It is against this backdrop that several studies [16, 17, 18, 19] have aimed to clarify the benefit of molecular profiling and subsequent targeted therapy, with variable results owing in part to the heterogeneity in clinical implementation of precision oncology. Here, we report the results of a retrospective study conducted on a Luxembourgish cohort of patients at a Molecular Tumor Board (MTB) with the aim of providing molecular diagnostics and targeted cancer therapy guidance.

## 2 Materials and methods

### 2.1 Patient selection

This study is a monocentric retrospective analysis of 94 patients affected by cancer who have undergone treatment at the Centre Hospitalier de Luxembourg (CHL). Molecular profiling with subsequent discussion in a specialized tumor board took place from 1 January 2018 to 31 December 2022. Eligible patients for the program were ≥18 years old with advanced solid tumors. All patients gave informed written consent for the use of their data for study and research purposes. Data extraction was performed anonymously through the CHL electronic patient medical record. Analysis time frame was set between July 2019 and March 2022 with a follow‐up cut‐off on 31 December 2022. This study received approval from the national research ethics committee (CNER) in accordance with the Declaration of Helsinki (license number 202207/06).

### 2.2 Targeted sequencing and molecular tumor board

Formalin‐fixed paraffin‐embedded (FFPE) tumor samples were sent to OncoDNA in Belgium for sequencing. The first six patients were analyzed through a 75 gene NGS panel while the following (88 patients) were evaluated through a broader 313 OncoDEEP gene panel after verification of adequate tumor cellularity of at least 10% by a qualified pathologist. OncoDEEP also provided data on Microsatellite Instability (MSI) and Homologous Recombination Deficiency (HRD) status as well as Tumor Mutational Burden (TMB). OncoDEEP is an ISO 15189 (Medical Laboratories‐Requirements for quality and competence), CE‐IVD (*In vitro* diagnostic devices complied to be sold in Europe), ISO 27001 (Information security management), and ISO 13485:2016 (Quality Management System) certified test, which is commercially available. Results were provided to the referring physician and the Institut National du Cancer (INC) for Molecular Tumor Board evaluation.

The dedicated MTB was organized by the INC as part of the first program for molecular diagnostics in Luxemburg (MDLUX2) and participants included an expert in medical and molecular oncology, a pathologist, a geneticist, and medical oncologists. The frequency of MTBs was approximately every 8 weeks and took place as a virtual meeting. The referring physician presented a brief patient history with past and present therapy lines, following which histological and genomic results were presented and discussed. A formalized report summarizing tumor cell percentage in the sample, found genomic alterations and therapy recommendations with OnkoKb actionability levels was established and provided to the referring physician after the MTB. Therapy recommendations had no prescriptive value and therapeutic decisions were left to the referring physician.

### 2.3 Statistical analysis

For the descriptive analysis, differences between matched and unmatched therapy patients was tested using Mann–Whitney‐U‐test for continuous or chi‐squared test for categorical data.

Patient characteristics have been described according to variable type, continuous variables with mean values, and qualitative variables with frequencies and percentages.

Progression‐free survival (PFS) of patients during a targeted therapy approach (PFS2) was compared with PFS of the previous treatment line of the same patients (PFS1). As such, patients were used as their own control for the main objective of this study. Besides PFS, overall survival (OS) and response variations (e.g., stable disease, complete response [CR] and partial response [PR]) are investigated. A PFS2/PFS1 ratio ≥1.3 was chosen as defining a benefit from targeted therapy in keeping with previous molecular‐matched treatment studies. Thus, the primary end point for this study was to establish the percentage of patients under targeted treatment with a PFS2/PFS1 ratio ≥1.3. As established in previous studies [17, 20], if the percentage of patients fulfilling that criterion exceeded 15%, molecularly guided therapy is to be considered as effective. Treatment response was assessed according to RECIST 1.1 criteria applied to patient imaging reports. Only patients who had a prior treatment line and were treated for at least 3 weeks were eligible for statistical analysis.

An exact binomial test was applied to investigate, if the percentage of patients with a PFS2/PFS1 ratio ≥1.3 was ≥15% using a one‐sided significance level of 5%. The Agresti–Coull 95% confidence interval for proportions was used for the probability of success.

A patient‐by‐patient bar plot is provided, showing PFS1 and PFS2 as bars for each patient in the targeted treatment group, ordered by descending PFS2/PFS1 ratio and including a horizontal line at ratio 1.3.

Additionally, Kaplan–Meier (KM) curves and risk tables were provided for PFS2/PFS1 ratio and OS for the matched treatment group with each a strata for targeted and non‐targeted patients and a KM plot of patients in the targeted treatment group with strata for PFS1 and PFS2. Comparisons between PFS1 and PFS2 Kaplan–Meier curves was assessed by applying a log‐rank test.

## 3 Results

### 3.1 Patient characteristics

A total of 94 patients were referred for a NGS panel test,10 patients were subsequently excluded because NGS could not be performed on the provided tumor sample. The results of the remaining 84 patients were then discussed at the MTB. Patient characteristics are summarized in Table 1. The most frequent tumor types were colorectal (*n* = 10), breast (*n* = 9), sarcoma (*n* = 8), pancreatic (*n* = 6), and uterine (*n* = 6). Patient characteristics were similar in the matched treatment and unmatched treatment group. Mean age was 54 years with a slight predominance of female patients (56%). The median number of previous therapies was 2, with 32% of patients having received more than two previous lines of therapy.

**Table 1:** Patient characteristics.

	Matched treatment *N* = 45	Unmatched *N* = 39 treatment group
Mean age at inclusion	53.38 years	54.54 years
Gender		
Female	28 (62.22%)	19 (48.72%)
Male	17 (37.78%)	20 (51.28%)
Tumor type		
Adrenal	0 (0%)	1 (2.56%)
Anal carcinoma	0 (0%)	1 (2.56%)
Breast	6 (13.33%)	3 (7.69%)
Cholangiocarcinoma	2 (4.44%)	3 (7.69%)
CRC	7 (15.56%)	3 (7.69%)
CUP	2 (4.44%)	1 (2.56%)
Gastric	3 (6.67%)	2 (5.13%)
Glioblastoma	3 (6.67%)	0 (0%)
H&N	0 (0%)	1 (2.56%)
Kidney	1 (2.22%)	1 (2.56%)
Kidney (bellini)	0 (0%)	2 (5.13%)
Liver	0 (0%)	2 (5.13%)
Lung	4 (8.89%)	0 (0%)
Melanoma	2 (4.44%)	1 (2.56%)
Merkel cell	0 (0%)	1 (2.56%)
Mucin rich appendix adc	0 (0%)	1 (2.56%)
Neuroendocrine	3 (6.67%)	0 (0%)
Ovarial	1 (2.22%)	0 (0%)
Pancreatic	3 (6.67%)	3 (7.69%)
Peritoneal PseudoMyxoma	1 (2.22%)	0 (0%)
Prostate	0 (0%)	2 (5.13%)
Renal	0 (0%)	0 (0%)
Sarcoma	2 (4.44%)	6 (15.38%)
Thyroid	0 (0%)	1 (2.56%)
Urological	2 (4.44%)	1 (2.56%)
Uterine cancer	3 (6.67%)	3 (7.69%)
Metastasis at time of inclusion		
No	6 (13.33%)	9 (23.08%)
Yes	36 (80%)	30 (76.92%)
Number of metastatic sides		
0	5 (11.11%)	1 (2.56%)
1	16 (35.56%)	16 (41.03%)
2	11 (24.44%)	12 (30.77%)
3	10 (22.22%)	6 (15.38%)
4	3 (6.67%)	3 (7.69%)
Previous therapy lines		
0	2 (4.44%)	6 (15.38%)
1	8 (17.78%)	11 (28.21%)
2	14 (31.11%)	13 (33.33%)
3	7 (15.56%)	4 (10.26%)
4	6 (13.33%)	2 (5.13%)
5	3 (6.67%)	0 (0%)
>5	3 (6.66%)	2 (5.13%)

### 3.2 NGS results and MTB recommendations

**Fig. 1:**
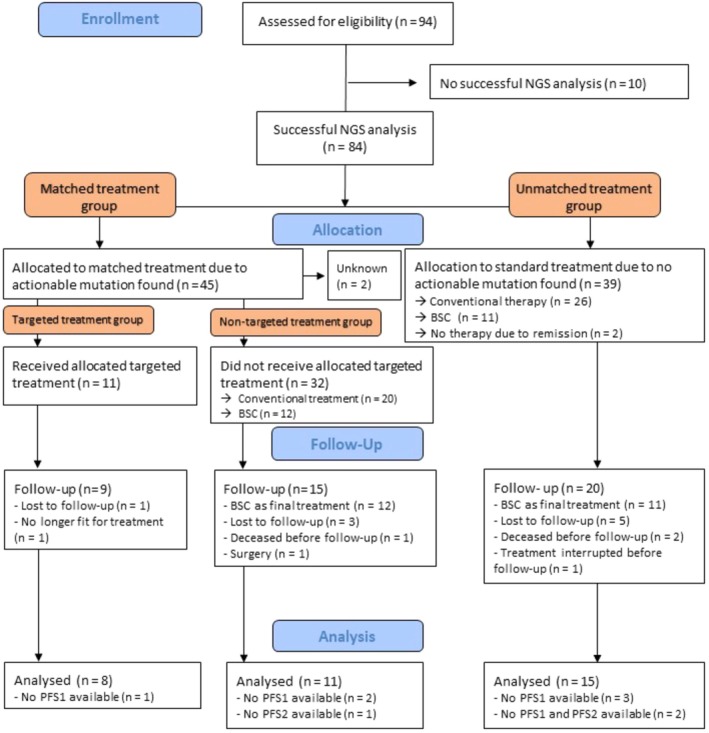
Study flow chart. In order to analyze the data patients were ordered into groups according to NGS results and whether they were treated with a targeted treatment or conventional treatment. NGS analysis was unsuccessful in 10 of 94 Patients. Actionable mutations were found in 45 of 94 patients. Eleven patients received targeted therapy with a possibility to calculate the PFS ratio for eight patients. BSC, Best supportive care; NGS, Next generation sequencing; PFS, Progression‐free survival.

**Fig. 2:**
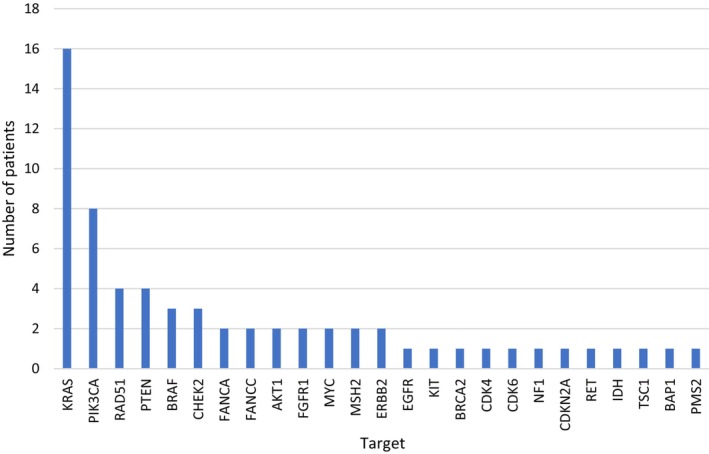
Genes with actionable mutations found in the study population. KRAS and PIK3CA Mutations were significantly more frequently found than other actionable mutations. Various gene mutations which have been linked with Homologous Recombination Deficiency (BRCA2, RAD51, CHEK2, FANCA, FANCC, BAP1) were identified. The cross‐entity nature of the study is reflected in the diversity of mutations.

The study flow is reported in Fig. 1. We recorded a NGS analysis failure rate of 10,6% with a median time from biopsy to molecular tumor board of 12.5 months. 45 (54%) patients were determined to have actionable alterations. Mutations in TP53 (30%), KRAS (19%), and APC (10%) were the most frequently observed. The most commonly altered genes that were deemed actionable were KRAS (mostly G12D), PIK3CA, RAD51, and PTEN (Fig. 2). Accordingly, the most recommended targeted treatments were MEK, PI3K, and PARP Inhibitors. Co‐occurring mutations that have been associated with therapeutic resistance (e.g., KRAS, TP53) did not preclude from targeted therapy assignment. OnkoKb lvl 4 (compelling biological evidence) was the most frequent evidence level for MTB recommendations. Regarding targets of patients for which targeted treatment recommendations were followed, six were evaluated as OnkoKb evidence level IV, two OnkoKb evidence level III, and three OnkoKb evidence level I.

In 11 out of 45 (24%) patients with actionable mutations, the MTB therapeutic recommendation was followed by the referring physician. Instead, 20 (44%) received conventional treatment and 12 (27%) received best supportive care. As a result, of all 94 patients that were initially included, ultimately 12% were treated with molecularly guided therapy.

### 3.3 Clinical outcomes

**Fig. 3:**
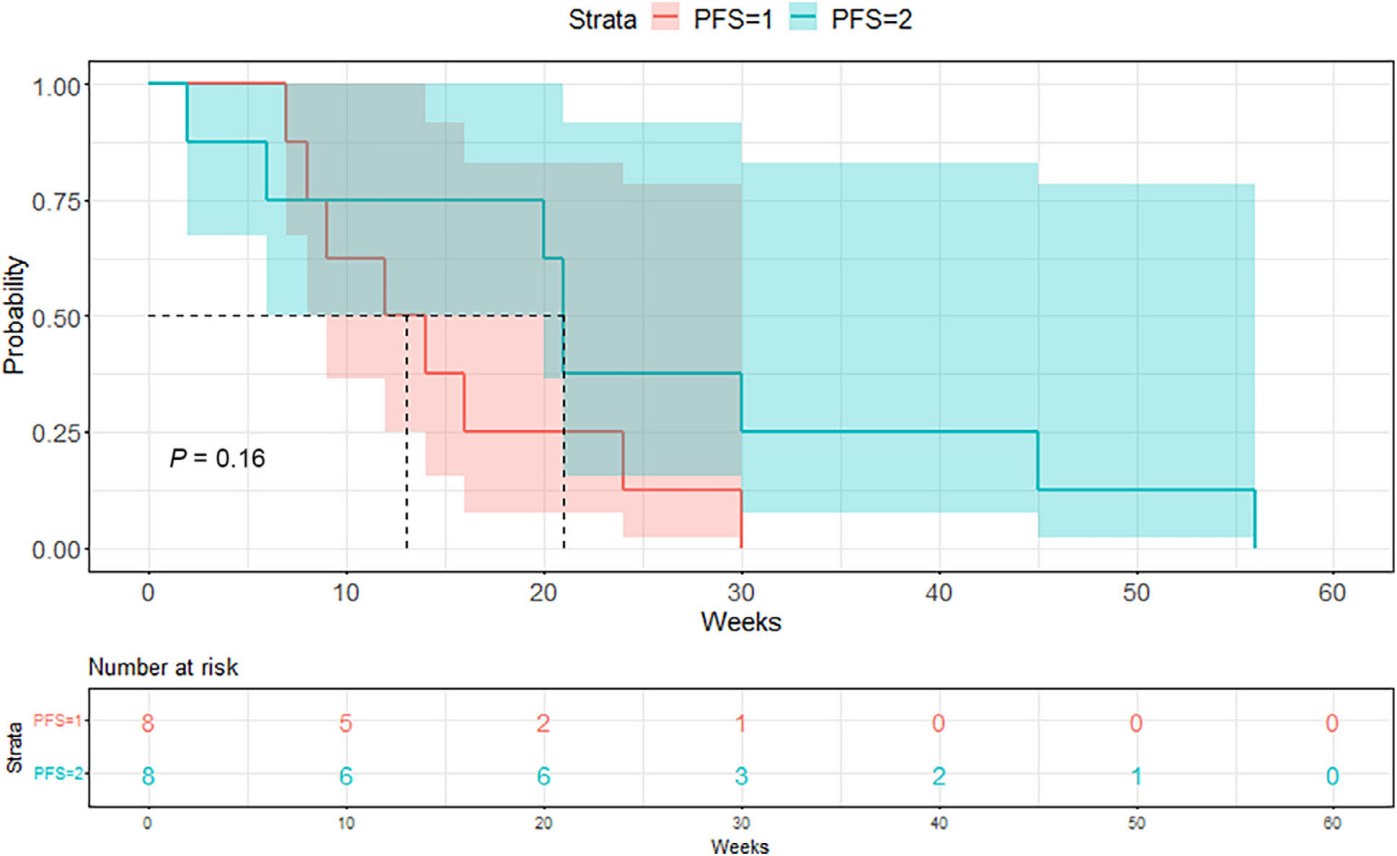
Kaplan–Meier curve of PFS1 and PFS2 for targeted treatment group and *P*‐value of log‐rank test. *P*‐value calculated by chi‐squared test. The Kaplan–Meier curves show a longer progression‐free survival with targeted therapy (PFS2) than with the previous conventional therapy (PFS1) among treated patients.

**Fig. 4:**
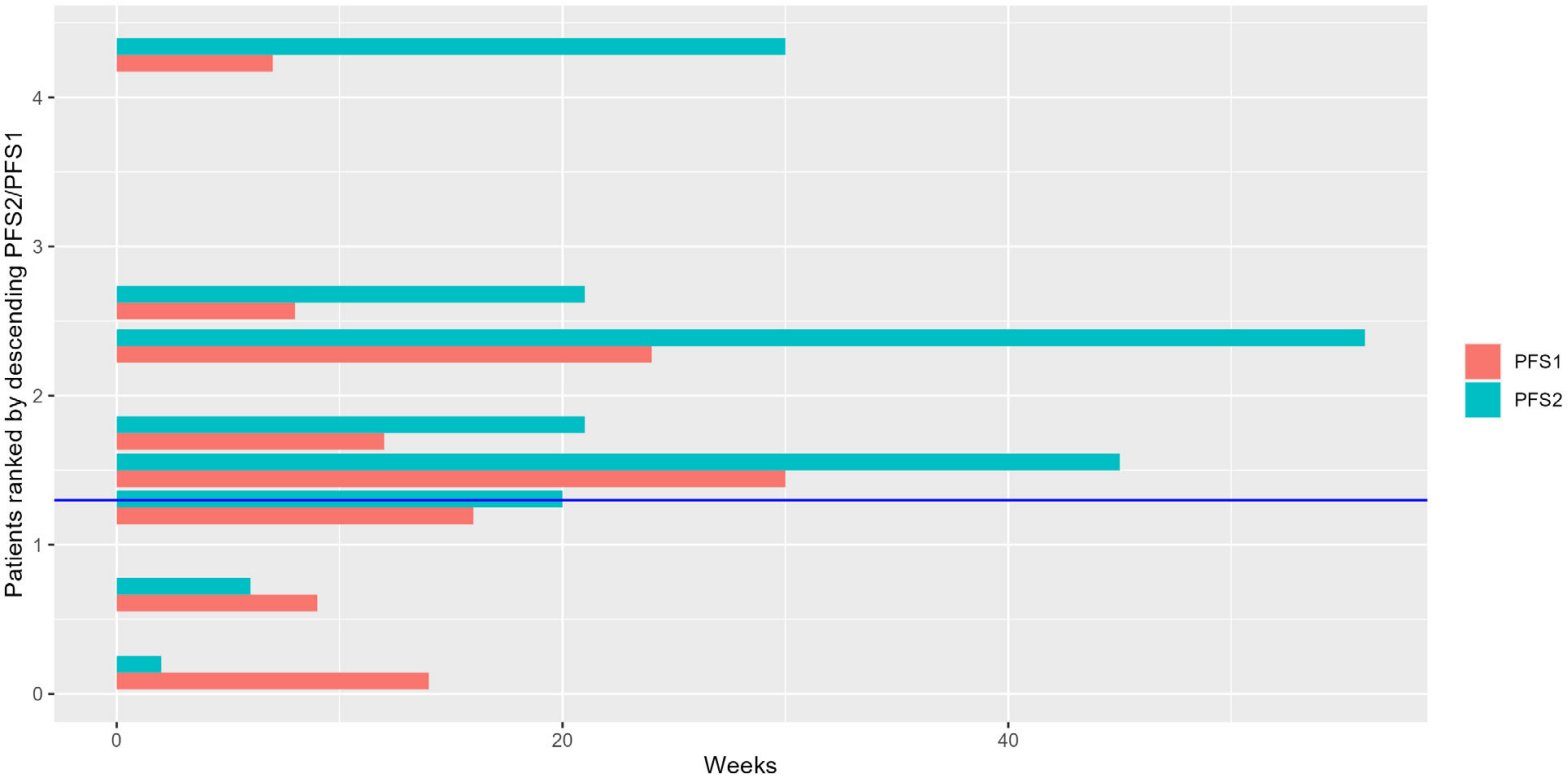
Individual PFS1 and PFS2 duration of targeted treatment patients, ordered by descending PFS2/PFS1 (*n* = 8). Patients above the blue horizontal line have PFS2/PFS1 > 1.3. Five of eight Patients had a progression‐free survival with targeted therapy (PFS2) which was more than 1.3 times longer than their previous conventional therapy (PFS1).

The primary end point could be evaluated in eight of 94 patients (one patient did not have an available PFS1, one patient was lost to follow up, and one patient was no longer fit to undergo treatment less than 2 weeks after therapy initiation). Patients who received a targeted treatment had a median PFS1 of 13 [9; NA] weeks and a median PFS2 of 21 [20; NA] weeks (Fig. 3). Patients who did not receive their proposed matched treatment had a median PFS1 of 25 [16; 76] weeks and a median PFS2 of 8 [4; NA] weeks. In the targeted treatment group, five patients of eight (62.5%), (Agresti–Coull 95% confidence interval [30.38; 86.51]) had a PFS2/PFS1 ratio >1.3 which is significantly higher than the assumed rate of 15% (*P*‐value: 0.0029) (Fig. 4). When the power calculation was performed, a PFS2/PFS1 ≥ 1.3 rate of 30% (alternative proportion) was assumed, which resulted in a power of 79.4% for a sample size of 47 patients. Reducing the sample size to *n* = 8 patients, the power of the test would be 19.41%, but as the estimated rate was higher than the assumed rate (62.5%), the sample size of eight patients has a power of 86.26% to show a significant difference to a rate of 15%. The null hypothesis of ≤15% of the patient population having a PFS ratio of ≥1.3 could therefore be rejected by our results, leading to our conclusion that patient outcome was improved by targeted therapy.

Interestingly, median PFS1 was shorter than median PFS2 in the targeted treatment group while median PFS2 was shorter than median PFS1 in the conventional therapy group (Fig. 3). According to RECIST 1.1 criteria, following targeted therapy, three patients showed a partial response (*n* = 2 BRAF inhibitor and MEK inhibitor association, *n* = 1 MEK inhibitor), four patients showed stable disease (*n* = 1 PARP inhibitor, *n* = 2 MEK Inhibitor, *n* = 1 immune checkpoint inhibitor), and one patient progressive disease (*n* = 1 mTOR inhibitor) at follow‐up (Table 2).

**Table 2:** Mutations, targeted treatment and best responses of the 8 patients with evaluable PFS1/PFS2 ratio. PD, progressive disease; PR, Partial response; SD, stable disease.

Mutation	Targeted treatment	Best response
BRAF V600E	BRAF inh. + MEK inh.	PR
BRAF V600K	BRAF inh. + MEK inh.	PR
KRAS G12V	MEK inh.	PR
RAD51 LOH	PARP inh.	SD
MSH2 (MSS)	Immune checkpoint inh.	SD
NF1	MEK inh.	SD
KRAS G12D	MEK inh.	PD
PIK3CA	PI3K inh.	PD

## 4 Discussion

The chosen primary end point of PFS2/PFS1 ratio as first proposed by Van Hoff [20], underpinned by the observation that PFS decreases as the number of lines of therapy progresses and widely used to the present purpose of determining treatment benefit, allowed us to further demonstrate the benefit of implementing targeted treatment by way of NGS and guidance of a dedicated MTB. As such, despite the limited number of patients included, this study of the implementation of molecular profile guided cancer therapy in Luxembourg adds to the growing evidence of the beneficial impact of targeted therapy on patient outcomes.

We noted that 27% of patients with actionable mutations received best supportive care instead of the MTB recommended targeted therapy suggesting that a non‐negligible proportion were no longer fit for treatment at the time of the MTB. Furthermore, median PFS1 before targeted treatment was remarkably short. As such we need to consider whether earlier molecular profiling might have benefited these patients since oncogenic driver targeting might have ameliorated clinical outcomes and allowed subsequent conventional therapies. In other words, the benefit shown by molecular guided therapy as applied in this Luxemburgish cohort might have been greater if molecular profiling was performed earlier in disease progression.

As expected, OnkoKb level I recommendations presented the best responses, with two of three OnkoKb level I treatments leading to partial responses. Comparable studies have found similar results [17, 21] although comparison is hampered by the use of divergent actionability scales. Discussions about how to best classify mutations according to therapeutical value are ongoing [22], with efforts to establish a common scale on an international level to guide clinical decisions leading to the proposal of the ESCAT framework [6], but national and regional regulatory approval processes have hampered such enterprises.

It must be noted that as evidence grows, MTB recommendations over these last few years have evolved accordingly. In this instance to illustrate this point, our study has found that most patients had treatment recommendations, which were graded as OnkoKb level IV and one of the most frequently recommended treatments was MEK inhibition for KRAS nonG12C mutations, both elements in all probability might not be representative of most current MTB recommendations in light of more recent evidence [23, 24]. As such, there is a constant need to update therapeutical proposals to keep up with nascent evidence.

Although precision medicine has been recognized as a valuable strategy in solid tumor therapy, divergent responses to known targeting oncogenic drivers point to an as of yet incomplete knowledge of molecular cancer processes. Consequently, broader molecular profiling programs, which include whole genome sequencing [25, 26], proteomics [27], transcriptomics [28], and more to characterize individual oncogenic processes, are establishing themselves to improve our understanding through expansive data collection, with promising results regarding patient outcomes. Another inevitable consequence of the growing amount of data available in this field is the necessity of a MTB composed by experts in the field of clinical oncology, genetics, and molecular biology to give therapeutic recommendations, which take into account complexities such as allele frequency [29], concomitant mutations [11], resistance mechanisms, and variant pathogenicity [30], and thus improve patient outcomes.

This study was limited mainly by its retrospective nature and by the small number of patients who underwent targeted treatment. Distinguishing the therapeutic value of individual genomic targets would have benefitted from a prospective study of clinical outcomes in a larger patient cohort, accordingly this study limited itself to evaluating the benefit of molecular profiling as implemented in Luxembourg at the CHL.

## 5 Conclusions

From these observations, we predict that at the current rate of development, targeted cancer therapy will rapidly take an ever‐greater role in cancer therapy, which further underlines the necessity of establishing adequate clinical implementation standards and processes in such a manner that patients continue to benefit from present and future advancements.

## Conflict of interest

The authors declare no conflict of interest.

## Author contributions

GB and MD conceived and designed the project; NG and MD acquired the data; AS and MD analyzed and interpreted the data, MD wrote the paper.

### Peer review

The peer review history for this article is available at https://www.webofscience.com/api/gateway/wos/peer‐review/10.1002/1878‐0261.13785.

## Data Availability

The data that support the findings of this study are available from the corresponding author (dangphuoc.michael@chl.lu) upon reasonable request.
